# Small bowel and lung histiocytic sarcoma revealed by acute peritonitis: A case report with review of literature

**DOI:** 10.1016/j.amsu.2021.102638

**Published:** 2021-07-27

**Authors:** Ahlem Bellalah, Ibtissem korbi, Seifeddine Ben Hammouda, Asma Achour, Nouha Ben Abdeljelil, Manel Njima, Amira Daldoul, Rim Hadhri, Leila Njim, Abdelfatteh Zakhama

**Affiliations:** a Department of Pathology, Fattouma Bourguiba University Hospital, Monastir, 5000, Tunisia; b Department of Digestive Surgery, Fattouma Bourguiba University Hospital, Monastir, 5000, Tunisia; c Department of Radiology, Fattouma Bourguiba University Hospital, Monastir, 5000, Tunisia; d Department of Carcinology, Fattouma Bourguiba University Hospital, Monastir, 5000, Tunisia; e Faculty of Medicine, University of Monastir, Monastir, 5000, Tunisia

**Keywords:** Histiocytic sarcoma, Small intestine, Lung, Acute peritonitis, Prognosis

## Abstract

**Introduction and importance:**

Histiocytic sarcoma (HS) is a rare malignant neoplasm showing morphologic and immunohistochemical features of histiocytes. It is characterized typically by extranodal presentation and a poor clinical course, particularly in cases with disseminated disease.

**Case presentation:**

This report documents a case of bifocal and aggressive HS in small bowel and lung revealed by acute peritonitis in a 63-year-old man.

**Clinical discussion:**

Despite its rarity, we believe that the correct diagnosis of HS is crucial for clinical treatment and prognostic prediction.

**Conclusion:**

The collection of additional cases of HS are important to obtain further progress in prognosis and guide treatment decisions.

## Highlights

• Histiocytic sarcoma is a rare malignant neoplasm showing morphologic and immunohistochemical features of histiocytes.
• The diagnosis of histiocytic sarcoma can be extremely challenging because histologic overlap with diverse mimics.
• Judicious application of immunohistochemical markers is crucial to confirm the diagnosis.
• The collection and evaluation of additional cases are pivotal to obtain further progress in prognosis and guide treatment decisions.

## 1 Introduction

Histiocytic sarcoma (HS) is a very rare neoplasm defined by the recent 2016 revision of the World Health Organization classification of Tumors of Hematopoietic and Lymphoid Tissues as a malignant neoplasm showing morphological and immunohistochemical features of mature tissue histiocytes [1]. Most cases present in extranodal sites most commonly the intestinal tract, skin, and soft tissue [[2], [3], [4]].The clinical presentation of this tumor vary from a solitary mass to a widely disseminated disease. HS is often diagnosed at an advanced clinical stage with aggressive clinical evolution [5,6]. It has a poor response to chemotherapy with a high mortality rate [7]. We report anew case of HS arising in the ileum and the lung occurring in a 61-year-old man with a review of literature to highlight its clinical, histopathological, and prognostic aspects. This work has been reported in line with the SCARE criteria [8].

## 2 Case report

**Fig. 1:**
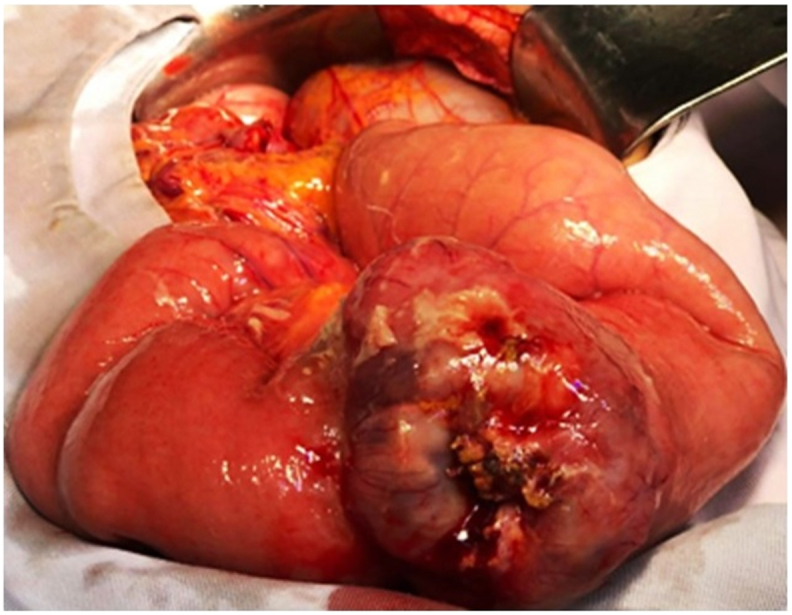
Intraoperative abdominal exploration showing a mass of the small bowel measuring 7 × 4 cm, complicated by perforation and adhering to the omentum.

**Fig. 2:**
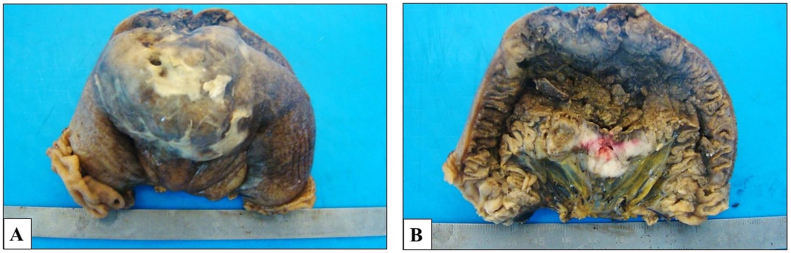
**A)** Gross examination of the specimen showing oval, ulcerative and infiltrative tumor perforating the intestinal wall. **B:** On cut section, the tumor was gray-white colored and contained foci of hemorrhage and necrosis.

**Fig. 3:**
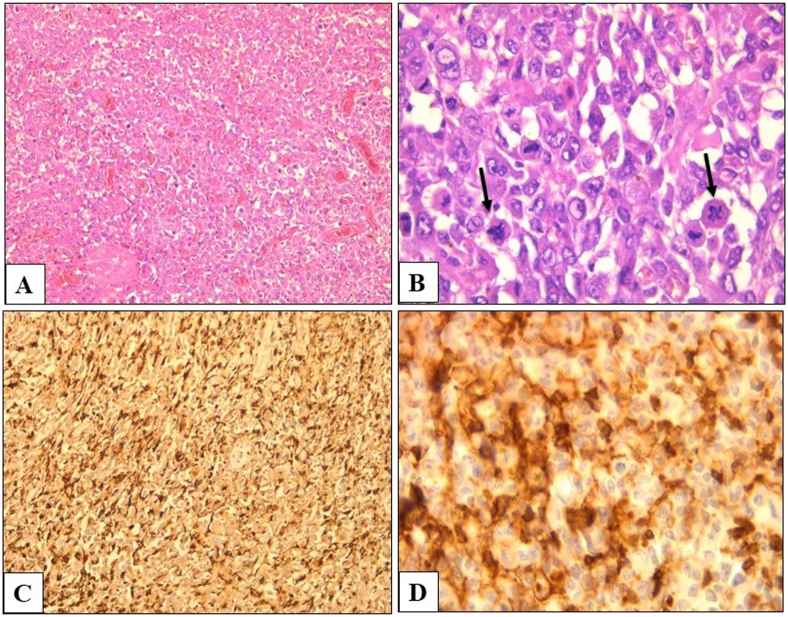
**A)** Sheets of poorly cohesive large cells arranged in nests and cords and separated by a rich vascular network (HE x 100). **B)** Pleomorphic tumor cells exhibiting numerous and abnormal mitosis (Black arrow) (HE x 400). Immunohistochemical stain shows diffuse immunoreactivity for CD68 (**C**) and CD45 (**D**).

**Fig. 4:**
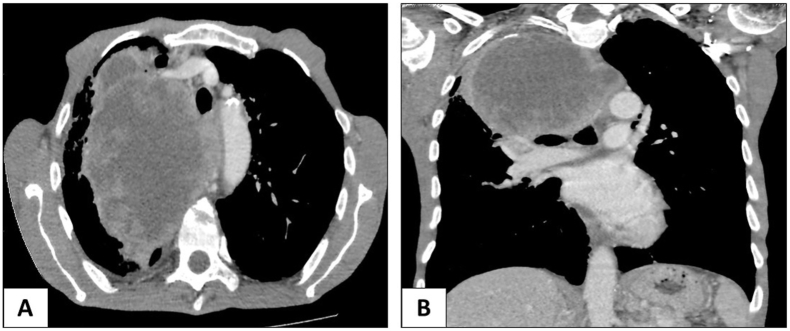
**A)** Chest CT scan in axial section showing right mediastino-pulmonary mass largely necrotic and measuring 13 cm in long axis. **B)** Coronal section showing that the mass pushes back the trachea and the esophagus and invades the carina.

This is a 63-year-old man without pathological history who presented to the Emergency Room for acute abdominal pain. Abdominal examination revealed a rebound tenderness suggesting for acute peritonitis. Abdominal Computed Tomography (CT) scan with contrast injection, showed a pneumoperitoneum with a perihepatic intraperitoneal effusion. Urgent surgical exploration was made by a university assistant in general surgery and found a purulent peritonitis with a mass of 7 × 4 cm of the small intestine complicated by perforation and adhering to the omentum (Fig. 1). A 20 cm resection of the small bowel was performed with a manual anastomosis. The postoperative course was uneventful. Macroscopic examination revealed an ulcerative and infiltrative tumor perforating the intestinal wall. On cut section, the tumor was gray-white colored and contained foci of hemorrhage and necrosis (Fig. 2). Histologically, it was composed of poorly cohesive large cells arranged in nests and cords separated by a rich vascular network and lymphocytes, plasma cells, benign histiocytes and eosinophils (Fig. 3, A). Tumor cells had pleomorphic nuclei and abundant eosinophilic cytoplasm. Mitotic figures were numerous and often abnormal (Fig. 3, B). On immunohistochemical study, tumor cells were diffusely positive for CD68 (Fig. 3, C) and CD45 (Fig. 3, D), focally positive for S-100 protein but negative for HMB45, Melan A, Dog1 and c-Kit. Immunostaining with epithelial, vascular, lymphoid, neuroendocrine markers was also negative. The diagnosis of histiocytic sarcoma of the ileum was made. Chest CT scan was made then for extension assessment and showed a right pulmonary mass measuring 13 × 4 cm with mediastinal invasion (Fig. 4). A lung biopsy was performed and confirmed the pulmonary involvement by HS. The patient was referred to the department of carcinology after spending one week at our hospital and he died before receiving chemotherapy.

## 3 Discussion

Histiocytic sarcoma is an extremely rare malignant neoplasm included within the tumors of the macrophage-dendritic cell lineage [1]. Its pathogenesis is still unclear. Some cases of HS arising by transdifferentiation from low-grade B-cell lymphoma or after chemotherapy for mediastinal or gonadic germ cell tumors are reported [8,9]. Most cases of HS occur in adults with a male predilection [2]. However, pediatric cases are reported. The most common presentation of histiocytic sarcoma is a painless solitary mass, which can be associated to fever, loss of weight, and night sweats [10]. Sites of involvement include usually the gastrointestinal tract, the spleen, the liver, soft tissue and rarely the skin, the lymph node and the brain [[2], [4], [12], [13]].

Clinical presentations of HS are variable, ranging from localized disease to widely disseminated disease with multifocal masses [2,4]. According to the literature, a subset of patients with HS has a history of hematolymphoid disorder such as chronic lymphocytic leukemia/small cell, follicular lymphoma, mantle cell lymphoma and multiple myeloma [[4], [11], [12], [13]].

On histology, the tumor consists of a diffuse non-cohesive proliferation of oval to round large cells (>20 p.m.). Tumor cells have usually an abundant eosinophilic cytoplasm and pleomorphic nuclei. The extent of mitotic activity parallels closely the degree of nuclear pleomorphism. Large multinucleated cells are commonly seen. Features of haemophagocytosis can be observed in the neoplastic cells. An intense and marked inflammatory infiltrate of small lymphocytes, neutrophils or eosinophils is often seen [1,2,4,6].

Immunohistochemistry is pivotal in the diagnosis of HS, to confirm histiocytic differentiation with positive markers and to exclude morphologic mimics with negative markers. Most HSs express CD68, CD163, PU.1and CD45 [[2], [4], [14], [15]]. It is recommended to have an expression of at least two of the following markers (CD163, CD68, CD4, and lysozyme) as a diagnostic criterion in histiocytic sarcoma [15]. Aside from these markers, HS can also variably express CD45, CD15, HLA-DR, factor XIIIa and S-100 [[4], [16], [17]]. The expression of CD45 is also recommended to establish hematopoietic origin and exclude morphologically similar soft-tissue neoplasms. Besides, HS is expected to be negative for melanocytic (SOX10, HMB-45, MART-1), epithelial (keratin, EMA), Langerhans cell (CD1a, langerin), myeloid cell (CD13, MPO), follicular dendritic cell (CD21, CD35), specific B-cell and T-cell (CD20, PAX5, CD3) and vascular (CD31, ERG) markers.

The differential diagnosis of HS is notably broad and includes other malignant neoplasms such as large-cell non-Hodgkin's lymphomas, metastatic carcinoma, malignant melanoma and soft-tissue sarcoma. Benign entities with histiocytic proliferation, like storage disorders and hemophagocytic syndrome can be excluded because of malignant cytologic features [2,4,18]. HS and hematolymphoid neoplasms appear clonally related because they share identical molecular alterations [19,20]. Molecular studies often showed monoclonal IgH gene rearrangements and reported some cases of HS with BRAF alteration, including V600E5 and non-V600E mutations [[19], [20], [21]].Immunoglobulin H (IgH)–BCL2 fusion has been detected in patients harboring histiocytic sarcoma, follicular lymphoma and chronic lymphocytic leukemia/small cell lymphoma [9,14,19].

The gold standard of treatment of HS is surgical resection with wide margins. Other treatment options include chemotherapy and radiotherapy in case of incomplete tumor resection [22]. The site of the tumor, the size and the stage of the disease are believed to be the most important factors in the prognosis [13]. The involvement of central nervous system and disseminated disease are usually aggressive with a clinical course ranging from 0.5 to 36 months despite complete surgical excision and chemotherapy [23,24].

## 4 Conclusion

HS is a very rare neoplasm with malignant proliferation of cells showing morphological and immunohistochemical features of mature tissue histiocytes. The diagnosis of histiocytic sarcoma can be extremely challenging because its rarity and histologic overlap with diverse mimics. Thus, the recognition of morphologic features, as well as judicious application of immunohistochemical markers is crucial to confirm the diagnosis. The collection and evaluation of additional cases are pivotal to obtain further progress in prognosis and guide treatment decisions of this aggressive disease.

## Provenance and peer review

Not commissioned, externally peer reviewed.

## Sources of funding

This is not applicable for our manuscript.

## Ethical approval

Exemption from ethnical approval.

## Consent

Written informed consent was obtained from the patient for publication of this case report and accompanying images. A copy of the written consent is available for review by the Editor-in-Chief of this journal on request.

## Author contribution

Ahlem Bellalah and Seifeddine Ben Hammouda: data analysis and writing the paper. Manel Njima and Nouha Ben Abdeljelil: bibliography, coordination and helped to draft the manuscript. Ibtissem Korbi, Asma Achour and Amira Daldoul: specimen contribution and data collection. Abdelfatteh Zakhama, Rim Hadhri and Leila Njim: revision.

## Trial registry number

This is not applicable to our case report.

## Guarantor

Dr. Seifeddine Ben Hammouda.

## Declaration of competing interest

None.
